# Maternal Mental Health under COVID-19 Pandemic in Thailand

**DOI:** 10.3390/ijerph19010347

**Published:** 2021-12-29

**Authors:** Wachiranun Sirikul, Krongporn Ongprasert, Chanodom Piankusol, Penprapa Siviroj

**Affiliations:** Department of Community Medicine, Faculty of Medicine, Chiang Mai University, Chiang Mai 50200, Thailand; wachiranun.sir@cmu.ac.th (W.S.); krongporn.o@cmu.ac.th (K.O.); chanodom.p@cmu.ac.th (C.P.)

**Keywords:** maternal mental health, COVID-19, lockdown impacts, activities during lockdown, illness experiences

## Abstract

Numerous nations have implemented lockdown measures in response to the COVID-19 pandemic. As a consequence of the lockdown on daily living, social participation, and health service accessibility, vulnerable people, for example, new mothers, may experience an increase in mental health problems. This cross-sectional survey was conducted to investigate the impact of the COVID-19 pandemic lockdown on Thai new mothers and the variables affecting their mental health. The survey data were collected from 903 Thai mothers with infants aged 0–12 months using an online platform and a face-to-face interview questionnaire survey between 17 July and 17 October 2020, during the first nationwide COVID-19 lockdown period. For the final analysis, there were 862 participants who completed all of the questions. The full exploratory analysis was performed by multivariable linear regression to identify the variables influencing maternal mental health. Our study demonstrated that new mothers reported feeling a high extent to some extent of worry (44.9%), increased appetite (40.4%), becoming easily annoyed or irritable (39.1%), and feeling down (33.5%), whereas 82.7% felt able to cope with the first lockdown situation. Practiced relaxation techniques were associated with positive maternal mental health (adjusted β = 1.05, 95% CI 0.57 to 1.52, *p* < 0.001). The perceived impact of the COVID-19 lockdown was on the household’s ability to pay for rent, to make mortgage payments (adjusted β = −1.59, 95% CI −2.87 to −0.36, *p* = 0.011), the household’s ability to pay for other essentials, such as utilities and medication (adjusted β = −1.99, 95% CI −3.16 to −0.81, *p* = 0.001), household crowding after lockdown (adjusted β = −3.46, 95% CI −4.86 to −2.06, *p* < 0.001), and not going outside or doing outdoor activities (adjusted β = −2.22, 95% CI −3.35 to −1.08, *p* < 0.001). These impacts were significantly associated with negative mental health. In conclusion, our results emphasize the critical need for continuous monitoring of maternal mental health and developing an effective response strategy and activity for promoting maternal mental health under the stress of repetitive lockdowns and increased economic pressures.

## 1. Introduction

Since the global COVID-19 pandemic in late 2019, many countries have implemented restrictive measures such as social distancing through lockdown regulations. Social distancing is a recommended prevention strategy for the general population. It includes keeping a physical distance between people (at least one meter) and reducing contact with contaminated surfaces while encouraging and maintaining social connections within families and communities. As disease transmission began to spread to community levels, a nationwide lockdown was implemented as a public health emergency response to break the chain of transmission through movement restrictions, stay-at-home measures, and the closure of essential services (e.g., public transportation, health, and social services) [1,2]. The first confirmed case of COVID-19 reported in Thailand was on 13 January 2020, after which it continued to spread. As of 21 October 2021, there were 1,821,579 cases of COVID-19 and 18,559 deaths (1.01%) in Thailand [3]. The first lockdown in Thailand was imposed by the government with a curfew for the whole population from 10 p.m. to 4 a.m. between 3 April and 15 June 2020 [4].

The consequences of COVID-19 lockdown have resulted in extensive disruption to daily life. In the US, adverse mental health problems have been associated with the COVID-19 outbreak [5]. Many people have been left feeling anxious or depressed, fearful of infection, and stressed, making the future seem uncertain [6,7,8,9,10]. Moreover, the impact of lockdown can include the loss of income and housing and reduced support from their family, peer group, and health personnel, all of which contribute to changes in people’s daily living and their mental health [11,12,13]. Worries resulting from the pandemic are to be expected and common, but nevertheless might cause suffering and impairment in social and occupational dimensions. In addition to this, the restrictive measures might have psychological effects, causing an emotional disturbance, anxiety, anger, depression, insomnia, and somatic signs and symptoms [14,15].

Pregnant and postpartum women are vulnerable to COVID-19 [16,17,18]. Many mothers increased their time for childcaring but had greater stress (42%) and increased frustration (50%) [19]. New mothers across the world were shown to be affected by the lockdown in multiple ways, as indicated by an increase in negative emotions and psychological symptoms, including feeling down, anxious or worried, lonely, and having depressive or anxiety symptoms [18,20,21].

To the best of our knowledge, there are no published studies assessing maternal mental health during the COVID-19 pandemic and lockdown in Thailand. Since established evidence points out that the COVID-19 pandemic and lockdown measures aggravate mental health problems in new mothers and their households, this study aims to investigate the impacts of the COVID-19 pandemic and the nationwide lockdown on household capabilities, daily living activities, and the mental health of new mothers living in Thailand. We also examined the association between potential influencing factors and maternal mental health in order to suggest future preventive strategies for maternal mental health.

## 2. Materials and Methods

### 2.1. Study Design and Participants

The cross-sectional study was conducted via a nationwide online survey and a face-to-face interview survey from 17 July 2020 to 17 October 2020, after the first nationwide COVID-19 lockdown period in Thailand (from 3 April to 15 June 2020) and before the second wave of the outbreak from 21 December 2020 to 11 February 2021. We recruited post-partum women over 18 years old with infants under age 12 months at the time of the survey, regardless of nationality, living in Thailand during the COVID-19 pandemic. Using an online survey enabled us to capture mothers’ experiences during the pandemic in a timely and remote manner and to reach mothers from all regions of Thailand. Additionally, face-to-face interviews were performed in Northern Thailand at secondary and tertiary public hospitals as well as private hospitals, to represent a diversity of maternal socioeconomic backgrounds. Details regarding sample size, participant recruitment, and data collection have been published elsewhere [13].

**Figure 1:**
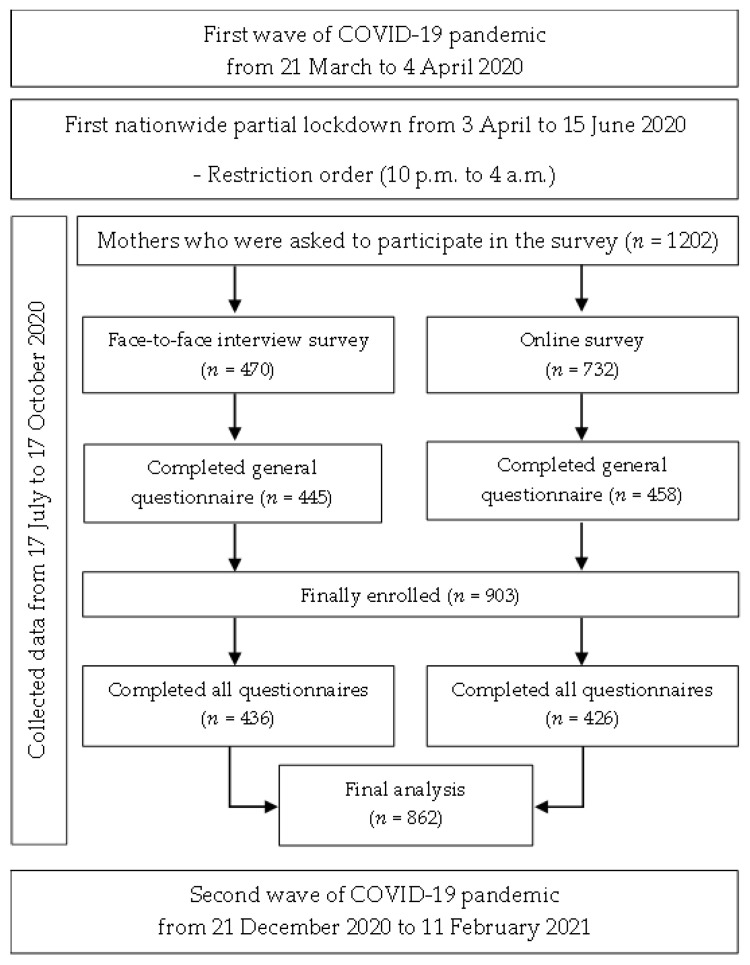
COVID-19 pandemic and lockdown durations in Thailand and period of the online platforms, interviews, and sample size of this study.

**Figure 2:**
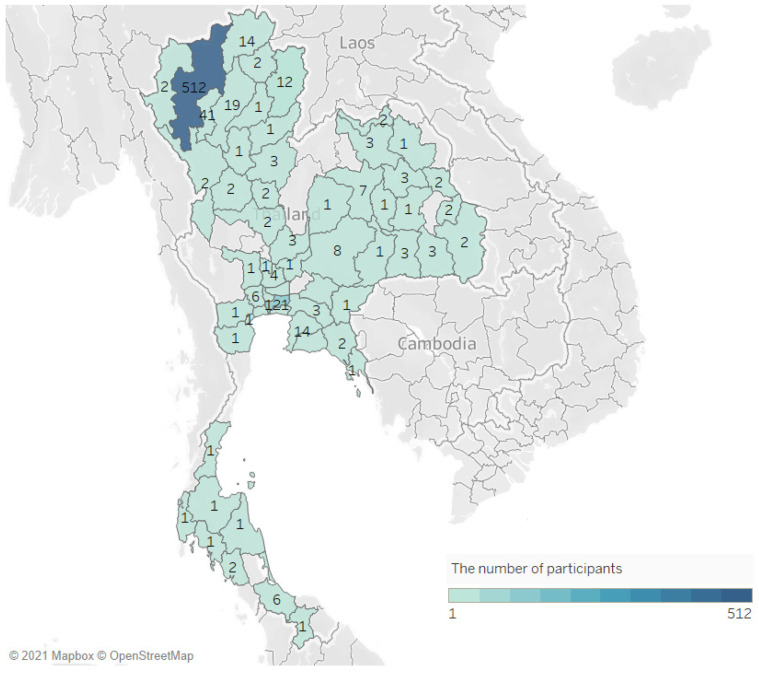
The distribution of survey participants. The figure was created by Tableau Desktop 2021.1, LLC, Seattle, WA, USA.

For the online survey, 732 participants gave their consent to participate in this study. Of those, 426 participants (58.2%) answered complete questions. The face-to-face interview survey had 436 (92.8%) participants out of 470 who answered complete questions. For the final analysis, the sample population consisted of 862 participants, recruited independently from the online platform (426, 49.4%) and face-to-face interviews (436, 40.6%) (Figure 1). The majority of participants lived in Chiang Mai (512, 56.6%) and Bangkok (121, 13.3%). Figure 2 illustrates the distribution of survey responses per province.

### 2.2. Questionnaire Design

The participants were asked about their experience of COVID-19 symptoms and if they had been tested for COVID-19, the mother’s activities, and moods during the COVID-19 lockdown. This study used a structured questionnaire adapted from the UK COVID-19 New Mum Survey in English [19] and translated into Thai; the pilot questionnaire was tested to improve the questions with 5 mothers at the well-baby clinic, Maharaj Nakorn Chiang Mai hospital before use. The questionnaire had the following five parts:

(1) Socio-demographic characteristics—age, ethnicity, marital status, education, employment status, household income, household members, type of accommodation (e.g., own house, apartment/condominium, dormitory, and rental house), number of rooms in the accommodation, and pets at home. Moreover, we collected the infant age, mother’s alcohol drinking during the last 7 days, and smoking in the last 2 days. The answers to these questions are “yes” or “no”.
(2) Experiences of COVID-19 symptoms and investigation—the questions in this section asked about the experience of COVID-19, if mothers and other members in the household had COVID-19 symptoms, and if the mothers had been investigated for COVID-19. The answers to these questions are “yes” or “no”.
(3) Perception of COVID-19 lockdown impacts—the questions in this section about how the lockdown impacted mothers’ life and activities in negative ways during the COVID-19 lockdown consisted of 6 questions. The answers to these questions were given on a 4-point Likert scale as major, moderate, minor, and no impact.
  (3.1) “Your employment/In what ways has your work been affected by COVID-19”.
  (3.2) “Your partner’s employment/In what ways has his work been affected by COVID-19”.
  (3.3) “Your household’s ability to pay for other essentials, such as utilities and medication”.
  (3.4) “Your household’s ability to pay for food”.
  (3.5) “Your household’s ability to pay for rent/to make mortgage payments”.
  (3.6) “Household crowding after lockdown”.
(4) Activities during the nationwide COVID-19 lockdown period—the questions in this section asked about the frequency of different activities, and the answers to these questions were given on a 4-point Likert scale as every day, more than 5 times per week, 4–5 times per week, 1–3 times per week, and never. These questions are in two parts, as follows:
  (4.1) Indoor/outdoor activities e.g., “went outside for a walk or for exercise”, “went shopping at the grocery store or pharmacy”, “participated in an online activity”, and “practiced a relaxation technique”.
  (4.2) Supportive activities e.g., “contact with a mother and baby support group or breastfeeding support group”, “contact with a health professional (general practioner (GP), health visitor, midwife), in person, by phone or online”, and “Attended an online, phone, or in person appointment with a mental health professional”.
(5) Maternal mental health—the questions in this section regarded the participants’ mood during the lockdown period. This tool was a 4-point Likert scale; to a high extent, to some extent, very little, and not at all. The overall score was calculated by the sum score of positive questions (no. 1–8) minus the sum score of negative questions (no. 9–18). This individual score will represent the maternal mood status and is used as the dependent variable in the exploratory analysis by Multivariable Linear Regression. The alpha coefficient for the reliability test of the mother’s mood questionnaire was 0.78 (Supplementary Table S1). There are two subsections of questions, positive and negative questions, as follows:
  (5.1) Positive ways had 8 items: “I’ve had the opportunity to chat with my family and friends”, “I feel connected with my local community”, “I’ve enjoyed the weather”, “I’ve had time to focus on my health”, “I’ve had time to exercise”, “I feel able to cope with the situation”, “I feel the house chores are more equally divided among household members”, and “I’ve had time to enjoy personal interests or hobbies”.
  (5.2) Negative ways had 10 items: “I’ve been having a poor appetite”, “I’ve been overeating”, “I’ve been feeling tired or having little energy”, “I’ve been feeling worried”, “I’ve been feeling down”, “I’ve had trouble falling or staying asleep”, “I’ve been feeling lonely”, “I’ve had trouble relaxing”, “I feel the house chores are less equally divided among household members”, and “I’ve become easily annoyed or irritable”.

### 2.3. Statistical Analysis

All statistical analyses were conducted using the STATA statistical software program (Stata Corp. 2019. Stata Statistical Software: Release 16, Stata Corp LLC, College Station, TX, USA). For categorical data, the maternal characteristics, perception of the lockdown impact, frequency of activities, and response to mother’s mood questions were reported as the frequency with percentages. The mean with standard deviation (SD) or the 95% confidence interval (95% CI) were used to present parametric data, while the median and interquartile range (IQR) were used to describe non-parametric data. The internal consistency and reliability of the maternal mental health questions were additionally evaluated by Cronbach’s alpha test. The association between maternal mental health (sum scores of the maternal mental health questions) during the nationwide lockdown and influencing variables, including maternal and family characteristics, the experience of COVID-19 symptoms and investigation, the perception of the lockdown impacts, and the activity during this period, was explored using multivariable linear regression. The directed acyclic graphs were designed to illustrate the set of confounders that needed to be adjusted and to identify the factors associated with the outcome from the regression model. A regression coefficient with 95% confidence intervals (CI) was reported to represent the magnitude of the association. The results of this study were reported according to the strengthening of the reporting of observational studies in Epidemiology (STROBE) checklist. All statistical analyses were two-sided, and a *p*-value of 0.05 was considered to be statistically significant.

### 2.4. Ethical Consideration

This study was conducted following the Declaration of Helsinki and the protocol was approved by the Research Ethics Committee, Faculty of Medicine, Chiang Mai University, Thailand (Study Code: COM-2563-07416).

## 3. Results

### 3.1. Characteristics of Participants

The characteristics of participants and their households are reported in Table 1. The participants consisted of 903 mothers with an infant aged less than one year old. Most of the participants were in the 19–35 years age group (78.1%) with an infant age of 6 months and younger (63.3%). Almost all were nuclear families (85.4%), had an employed occupation (73.3%), and had their own house (80.0%). About half of the mothers had education levels under a bachelor’s degree (69.5%) and 39.8% had pets at home. A few mothers and their family members had symptoms of COVID-19 (0.9% and 0.5%, respectively), while 7.6% of the participants were tested for COVID-19.

### 3.2. Mothers’ Perception of Impacts of COVID-19 Lockdown

Table 2 shows the mothers’ perceptions about the impact of the nationwide COVID-19 lockdown. Mothers described their perception of the effects of COVID-19 as moderate to major most frequently for “your employment/in what ways has your work been affected by COVID-19” (47.1%), “your household’s ability to pay for other essentials, such as utilities and medication” (45.0%), “your partner’s employment/in what ways has his work been affected by COVID-19” (43.7%), and “your household’s ability to pay for food” (42.6%) and less frequently for “your household’s ability to pay for rent/to make mortgage payments” (28.9%) and “household crowding after lockdown” (14.7%).

### 3.3. Mothers’ Activities during the COVID-19 Lockdown Period

As indicated in Table 1, most mothers had gone outdoors or had outdoor activities (63.8%), which included going outside to a public space (15.9%) and going to an outdoor space within the residence area (47.8%). Table 3 shows the proportion of mothers’ activities during the COVID-19 lockdown period. Considering the mothers’ activities, “participated in an online activity” (41.3%), “went outside for a walk or for exercise” (30.7%), and “practiced a relaxation technique” (28.8%) were most frequently rated as more than 5 times or everyday/4–5 times per week. The most frequent rating of “never to do” regarding health supportive activity from others such as health professionals and support groups were “appointment with a mental health professional” (96.9%), “contact with a health professional (GP, health visitor, midwife), in person, by phone or online” (77.6%), and “contact with a mother and baby support group or breastfeeding support group” (60.9%), respectively.

### 3.4. The Maternal Mental Health during the COVID-19 Lockdown Period

**Figure 3:**
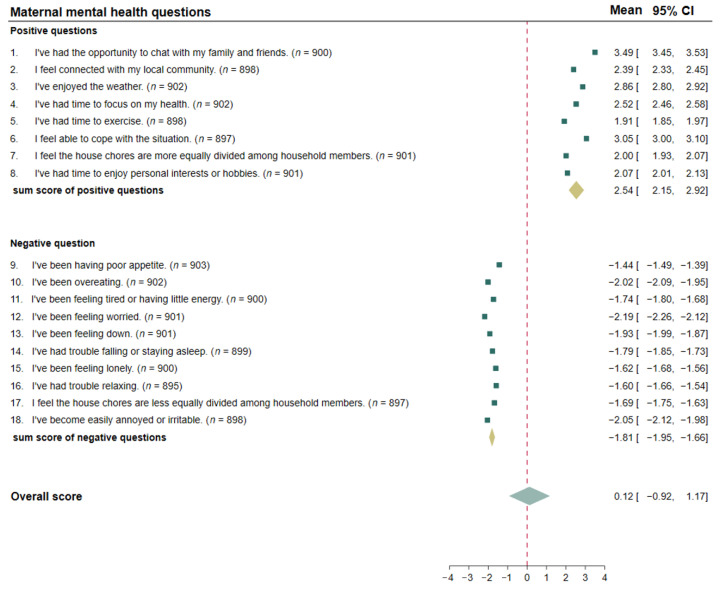
The estimated scores of the responses to the maternal mental health questions by forest plot.

In Figure 3, the forest plot summarizes the participants’ responses to the maternal mental health questions during the nationwide COVID-19 lockdown period. The first section of questions 1–8 indicated a positive mood of the mother and questions 10–18 reflected mental health from a negative perspective. The majority of mothers had a high to some extent of feelings about the opportunity to chat with family and friends (92.0%), being able to cope with the situation (82.7%), and appreciating the weather (74.0%) contributed to their positive mood. In contrast, more than half of the participants responded not at all or very little about the equal distribution of house chores (63.8%) and having time to enjoy personal interests or hobbies (63.6%). Nevertheless, more than half of the individuals reported none or very few negative physical symptoms or moods during the lockdown. The most frequent ratings regarding a high extent or some extent of negative moods were worrying (44.9%), increasing appetite (40.4%), becoming easily annoyed or irritable (39.1%), and feeling down (33.5%). Details of the mother’s mood responses are presented in Table S2.

### 3.5. The Associated Factors of Maternal Mental Health during the COVID-19 Lockdown Period

**Figure 4:**
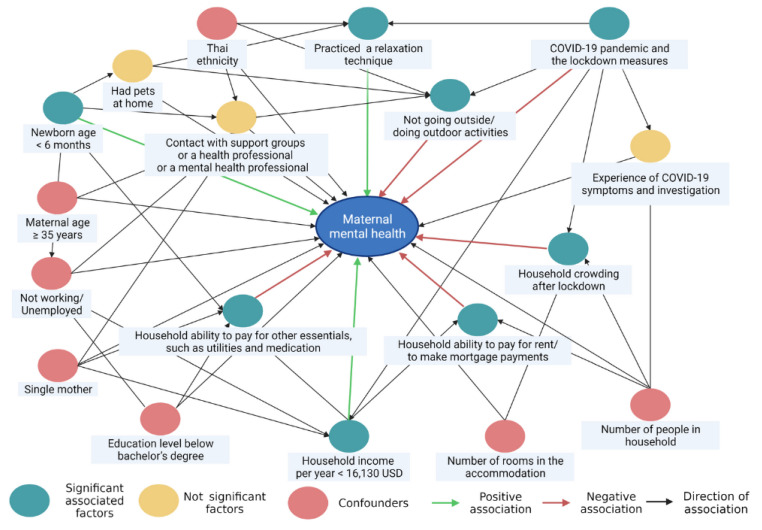
Relationship identification of maternal mental health during the COVID-19 nationwide lockdown period. Green ovals, yellow ovals, and pink ovals represent significant, not significant associated factors, and confounders, respectively. Green arrows indicate significant positive associations, whereas red arrows represent significant negative associations. Black arrows represent the direction of associations, which are not significant paths or confounding paths. The directed acyclic graphs were generated with publication licensed by BioRender, Toronto, Canada (Agreement number: QG237SORHP, 19 November 2021).

The results indicated mothers who had newborns aged less than six months, a household income per year of less than 16,130 USD, and practiced a relaxation technique had a significant positive effect on mood during the COVID-19 lockdown period, when other variables were controlled. On the other hand, the perceived impact of COVID-19 lockdown on “household ability to pay for rent/to make mortgage payments”, “household ability to pay for other essentials, such as utilities and medication”, “household crowding after lockdown”, and “not going outside or doing outdoor activities” had a significant negative effect on maternal mental health during the COVID-19 lockdown period after being adjusted with the other variables (Table 4). The possible relationship between maternal mental health and influencing factors as a consequence of the COVID-19 lockdown is illustrated in Figure 4.

## 4. Discussion

Maternal mental health problems are considered a major public health challenge and are recognized globally to have potential long-term consequences for mothers and infants [22]. During the first 12 months after childbirth, approximately 20% of mothers in developing countries and 13% in Southeast Asian countries have clinical depression [23]. Additionally, postpartum women frequently experience other mental health problems, such as anxiety, insomnia, and psychological distress [24,25]. Recent cohort and meta-analysis studies showed that the prevalence of maternal depression and anxiety increased during the COVID-19 pandemic compared with previous estimates [26,27]. As reported, the nationwide lockdown, resulting in a disruption of healthcare services and social interaction, has aggravated the mental health problems of new mothers. Our study demonstrated that new mothers reported their feelings as ‘high to some extent’ most frequently for worrying (44.9%), increased appetite (40.4%), becoming easily annoyed or irritable (39.1%), and feeling down (33.5%), which were higher than those reported in studies before the pandemic [24,25,28]. Given the fact that the majority of new mothers (82.7%) felt able to cope with their first lockdown situation, increasing awareness and support for maternal mental health should be advocated for to prevent this problem under the stress of repetitive lockdowns and outbreaks. This study aimed to explore the relationship between possible influencing factors and maternal mental health during the COVID-19 lockdown. According to the results, a quarter to half of the participant households had experienced significant impacts from the nationwide lockdown measures. Perceived moderate to major impacts on household overcrowding and households’ ability to make payments of household expenses showed an independent negative effect on maternal mental health. Furthermore, not going outside or doing outdoor activities was significantly related to poor mental health. Being in a low-income household, having an infant under six months, and more frequent practice of a relaxation technique were independent positive predictors of maternal mental health.

The possible explanation for a positive effect of having an infant under six months might be related to the timing of maternity return to work in Thailand according to the Labour Protection Act and Thailand culture [29]. The return-to-work study in Thailand showed that new mothers intended to return to work within 6 months due to the 98-day maternity leave policy with full pay for employees covered by the Social Security Scheme, economic concerns, and fear of losing their job. Some of the traditional Thai postpartum practices, including food restrictions, activity restrictions, and staying at home, may also influence their decision to return to work since these practices are still common in modern Thai culture [30]. Furthermore, mothers working in the informal private sector were not covered by maternity leave policies and those in lower-level positions were particularly vulnerable [31]. Mothers returning to work face several obstacles, from the responsibilities of both caring for their children and work stresses, particularly in the context of an economic downturn caused by the COVID-19 pandemic. It was surprising that low-income was independently associated with positive maternal mental health, not only in our study, but also the study in the US that reported living in lower socioeconomic status expressed improved postpartum mood over the lockdown period [32]. Moreover, having a low income is a risk factor for maternal mental health as reported in the previous studies during the lockdown period [18,20,21]. The explanation of this finding in our study is uncertain. It is possible that the pandemic and the first lockdown measures had a limited effect on low-income households or a greater effect on middle- to high-income households in our setting.

Consistent with other studies, difficulties in paying general household expenses were significantly associated with negative maternal mental health in the lockdown situations. Nevertheless, trouble with the ability to afford food during the lockdown, which negatively affected mental health in UK new mothers [20], was not a significant factor in our population and the Chinese population [21]. These disparities may be explained by differences in social contexts between Asian countries and the United Kingdom, as well as by government-supportive measures. The perception of household crowding after the lockdown had a substantial negative effect on maternal mental health independently and did not directly depend on the number of rooms or members in the household. This result was also observed in previous studies either before or during the lockdown. This emphasizes that the role of partners and family in helping new mothers is not only in providing a personal space within the household, but also in the emotional support offered. Our study also highlighted that practicing relaxation techniques was a significant positive factor in maternal mental health during the lockdown, whereas a negative association was found between not going outside or doing outdoor activities and maternal mental health. The benefit of practicing relaxation techniques in reducing anxiety was also found in a recent study during the lockdown [33]. Furthermore, a recent review of clinical studies discovered that there was a preventive effect of practicing relaxation techniques on postpartum mood disturbances [34]. The potential benefits of relaxation techniques and enhanced outdoor activity on maternal mental health and promoting these activities in new mothers during the lockdown period should be advocated. Additional investigations are necessary to conclude the actual benefits regarding the type, frequency, and duration of activity for the particularistic population.

The COVID-19 pandemic and lockdown have disrupted healthcare and social interactions, resulting in a loss of health service contact, a lack of support from health experts, and loss of social participation. In this study, remote contacting support groups (e.g., a mother and baby support group) and healthcare professionals were investigated as potential activities used to cope with the lockdown impact. Nevertheless, there was no significant association between having contact with support groups or healthcare practitioners and maternal mental health in our study, whereas improved mental health was related to these activities in the China and UK studies. This different result might be explained by the population preferences and available healthcare services in the different countries. Since most mental health services are only available in secondary and tertiary healthcare settings in Thailand, remote contact with healthcare professionals via phone calls or online platforms for mental health issues is not a common practice and was not widely available during the first lockdown. Furthermore, our data were collected during the first lockdown in situations in which the COVID-19 was generally under control and the lockdown was temporary. As a result, these activities may have a limited impact on maternal mental health in our study. Further studies should be supported in view of the recent situation (following the third and fourth waves of the COVID-19 pandemic) to determine an effective response strategy and activity for promoting maternal mental health under the stress of repetitive lockdowns and increased economic pressures.

Many established studies have shown that mothers with newborns are more likely to suffer from higher stress, worries, anxiety, and depression during the postpartum period, particularly in the COVID-19 pandemic. Our study provided evidence of exacerbated mental health and determined its associated factors in Thai new mothers during the first lockdown period. Nonetheless, our study has several limitations that should be addressed. The survey data were collected promptly from a large group of mothers one month after the first lockdown ended and before the second wave of the COVID-19 pandemic in Thailand using both online and face-to-face interview surveys. It is possible that recall bias may exist because of the timing of the survey; however, using an online survey and face-to-face interviews may be an effective method for recruiting participants who represent a diversity of maternal socioeconomic backgrounds. Our study participants might not represent mothers with poor socioeconomic levels and lack of social support who are unable access to healthcare services and the internet (e.g., minority groups, hill-tribes, and migrants). Moreover, the limitation of the cross-sectional design should be considered when interpreting the reported association between maternal mental health and the associated factors. Because these variables were examined concurrently, the temporal relationship between them cannot be determined. Another limitation is that rates of worry or anxiety in new mothers during lockdown could not be compared to pre-pandemic rates since mental health problems were not routinely evaluated in our settings.

## 5. Conclusions

In conclusion, our survey of Thai new mothers indicated that mental health problems increased during the first COVID-19 pandemic lockdown compared to the pre-pandemic prevalence. Practiced relaxation techniques were found to have a beneficial effect on maternal mental health. Encouraging new mothers to go outdoors or participate in outdoor activities during the pandemic should be weighed against the risk of infection and the positive effects on their mental health. The negative effects of household overcrowding and difficulties in paying for general household expenses as a result of the lockdown necessarily require additional support, particularly under the stress of repetitive lockdowns and increased economic pressures.

## Figures and Tables

**Table 1:** The characteristics of participants and households.

Characteristics	Total (*n* = 903)	
	*n*	%
Maternal age		
≤18 years	2	0.2
19–35 years	705	78.1
>35 years	196	21.7
Infant age		
≤6 months	572	63.3
6–12 months	331	36.7
Marital status		
Single mother	132	14.6
Nuclear family	771	85.4
Education		
Below bachelor’s degree	628	69.5
Bachelor’s degree or above	275	30.4
Employment		
Not working/Unemployed	80	11.4
Employed	514	73.3
Business owner	107	15.3
Household members, mean ± SD	3	±1
Type of accommodation		
Own house	722	80.0
Apartment/Condominium	46	5.1
Dormitory	114	12.6
Rental house	21	2.3
Number of rooms in the accommodation, mean ± SD	6	±2
Living conditions		
Can access to a private space/garden for doing an activity	423	47.8
Can access to a community space/garden for doing an activity	144	16.0
Not going outside/Doing outdoor activities	327	36.2
Household income per year		
Less than USD 16,130	314	34.9
More than USD 16,130	588	65.1
Had pets at home	359	39.8
Alcohol drunk during the last 7 days	18	2.0
Smoking in the last 2 days	2	0.2
Experience of COVID-19 symptoms and investigation		
Had symptoms	8	0.9
Any other member of household had symptoms	5	0.5
Had been tested for COVID-19	69	7.6

SD = Standard deviation.

**Table 2:** The perception of the nationwide COVID-19 lockdown impacts.

COVID-19 Lockdown Impacts (*n* = 903)	Levels of Impact			
	Moderate to Major		No or Minor	
	*n*	%	*n*	%
Your employment/In what ways has your work been affected by COVID-19	425	47.1	478	52.9
Your household’s ability to pay for other essentials, such as utilities and medication	406	45.0	497	55.0
Your partner’s employment/In what ways has their work been affected by COVID-19	395	43.7	508	56.3
Your household’s ability to pay for food	385	42.6	518	57.4
Your household’s ability to pay for rent/To make mortgage payments	261	28.9	642	71.1
Household crowding after lockdown	133	14.7	770	85.3

**Table 3:** Mothers’ activities during the nationwide COVID-19 lockdown period.

Activities (*n* = 903)	Levels of Activity (Times per Week), *n* (%)			
	>5 Times/Every Day	4–5 Times	1–3 Times	Never
Indoor/outdoor activity				
Participated in an online activity	283 (31.3)	90 (10.0)	182 (20.2)	348 (38.5)
Went outside for a walk or for exercise	172 (19.1)	105 (11.6)	309 (34.3)	316 (35.0)
Practiced a relaxation technique	160 (17.8)	99 (11.0)	282 (31.3)	359 (39.9)
Went shopping to the grocery store or pharmacy	67 (7.4)	101 (11.2)	501 (55.5)	233 (25.8)
Supportive activity				
Contact with a mother and baby support group or breastfeeding support group	83 (9.2)	58 (6.4)	211 (23.4)	548 (60.9)
Contact with a health professional (GP, Health Visitor, Midwife), in person, by phone or online	4 (0.4)	17 (1.9)	180 (20.0)	698 (77.6)
Attended an online, phone, or in person appointment with a mental health professional	0 (0.0)	6 (0.7)	22 (2.4)	871 (96.9)

GP = General practitioner.

**Table 4:** Association between socio-demographics, perception of COVID-19 lockdown impacts, maternal activities, illness experience, and maternal mental health.

Variables	β-coef.	95% CI	*p*-Value
Age ≥ 35 years	−0.29	−1.40 to 0.82	0.607
Newborn age < 6 months	1.14	0.13 to 2.15	0.026 *
Thai ethnicity	−0.98	−2.91 to 0.94	0.317
Single mother	0.10	−1.3 to 1.49	0.891
Education level below bachelor’s degree	0.85	−0.47 to 2.17	0.205
Not working/Unemployed	−1.33	−3.09 to 0.43	0.139
Household income per year < 16,130 USD	2.59	1.45 to 3.73	<0.001 **
Number of rooms in the accommodation	−0.10	−0.27 to 0.08	0.272
Number of people in household	0.38	−0.06 to 0.82	0.091
Had pets at home	−0.40	−1.42 to 0.62	0.439
Experience of COVID-19 symptoms and investigation			
Had symptoms	0.22	−5.12 to 5.56	0.936
Any other member of household had symptoms	−0.05	−6.4 to 6.3	0.988
Had been under investigated for COVID-19	−0.42	−2.24 to 1.4	0.653
COVID-19 lockdown impacts (moderate to major impacts)			
Household’s ability to pay for rent/to make mortgage payments	−1.59	−2.81 to −0.36	0.011 *
Household’s ability to pay for food	−0.52	−1.7 to 0.67	0.392
Household’s ability to pay for other essentials, such as utilities and medication	−1.99	−3.16 to −0.81	0.001 **
Impact on your employment/In what ways has your work been affected by COVID-19	−0.32	−1.37 to 0.74	0.554
Impact on your partner’s employment/In what ways has their work been affected by COVID-19	−0.54	−1.57 to 0.49	0.305
Household crowding after lockdown	−3.46	−4.86 to −2.06	<0.001 **
Not going outside/doing outdoor activities	−2.22	−3.35 to −1.08	<0.001 **
Activity during COVID-19 lockdown (times/week)			
Went shopping to the grocery store or pharmacy	−0.16	−0.75 to 0.43	0.591
Went outside for a walk or for exercise	−0.06	−0.54 to 0.42	0.796
Participated in an online activity	−0.21	−0.6 to 0.18	0.291
Contact with a mother and baby support group or breastfeeding support group?	0.01	−0.98 to 1	0.980
Contact with a health professional (GP, Health Visitor, Midwife), in person, by phone or online	−0.21	−0.74 to 0.32	0.443
Attended an online, phone, or in person appointment with a mental health professional	−1.48	−3.96 to 1	0.241
Practiced a relaxation technique	1.05	0.57 to 1.52	<0.001 **
Constant	5.33	−10.98 to 21.63	0.521

β-coef. = Beta co-efficient; GP = General practitioner; 1.00 USD = 32.00 THB; Analyzed by multiple linear regression; * Significant association at *p* < 0.05, ** Significant association at *p* < 0.001.

## Data Availability

The data presented in this study are available on request from the correspondent author.

## Supplementary Materials

The following supporting information can be downloaded at: https://www.mdpi.com/article/10.3390/ijerph19010347/s1, Table S1: Maternal mental health questionnaire reliability assessment, Table S2: Maternal mental health during the nationwide COVID-19 lockdown period.
