# Supplementary material for: Maternal Mental Health under COVID-19 Pandemic in Thailand
Source: Int J Environ Res Public Health. 2021 Dec 29;19(1):347. doi: 10.3390/ijerph19010347 (PMC8744545; doi:10.3390/ijerph19010347)
Supplement: Supplementary file 1 [file ijerph-19-00347-s001.zip › ijerph-1492348-supplementary.pdf]

Table S1. Maternal mental health questionnaire reliability assessment.

Questions	Sign	Item-Test Correlation	Item-Rest Correlation	Average Interitem Correlation	Cronbach's α	
						(If Item Dropped)
1. I've had the opportunity to chat with my family and friends.	+	0.31	0.19	0.18	0.78	0.70
2. I feel connected with my local community.	+	0.37	0.26	0.17	0.78	0.65
3. I've enjoyed the weather.	+	0.43	0.33	0.17	0.78	0.62
4. I've had time to focus on my health.	+	0.38	0.27	0.17	0.78	0.61
5. I've had time to exercise.	+	0.37	0.26	0.17	0.78	0.66
6. I feel able to cope with the situation.	+	0.48	0.38	0.17	0.77	0.59
7. I feel the house chores are more equally divided among household members.	+	0.10	−0.02	0.19	0.80	0.65
8. I've had time to enjoy personal interests or hobbies.	+	0.27	0.16	0.18	0.79	0.65
9. I've been having poor appetite.	−	0.48	0.37	0.17	0.77	0.60
10. I've been overeating.	−	0.32	0.21	0.18	0.78	0.65
11. I've been feeling tired or having little energy.	−	0.60	0.51	0.16	0.76	0.66
12. I've been feeling worried.	−	0.62	0.53	0.16	0.76	0.60
13. I've been feeling down.	−	0.58	0.49	0.16	0.76	0.64
14. I've had trouble falling or staying asleep.	−	0.67	0.59	0.15	0.76	0.60
15. I've been feeling lonely	−	0.66	0.58	0.16	0.76	0.60
16. I've had trouble relaxing.	−	0.43	0.32	0.17	0.78	0.61
17. I feel the house chores are less equally divided among household members.	−	0.63	0.55	0.16	0.76	0.60
18. I've become easily annoyed or irritable.	−	0.31	0.19	0.18	0.78	0.70
Test scale				0.17	0.78	

Table S2. Maternal mental health during the nationwide COVID-19 lockdown period.

Questions	Valid (*n*)	*n* (%)			
		Not At All	Very Little	To Some Extent	To A High Extent
1. I've had the opportunity to chat with my family and friends.	900	9 (1.0)	64 (7.1)	301 (33.5)	526 (58.5)
2. I feel connected with my local community.	898	220 (24.5)	202 (22.5)	384 (42.8)	92 (10.2)
3. I've enjoyed the weather.	902	87 (9.7)	147 (16.3)	473 (52.4)	195 (21.6)
4. I've had time to focus on my health.	902	143 (15.9)	260 (28.8)	379 (42.0)	120 (13.3)
5. I've had time to exercise.	898	393 (43.8)	235 (26.2)	225 (25.0)	45 (5.0)
6. I feel able to cope with the situation.	897	30 (3.3)	125 (14.0)	505 (56.3)	237 (26.4)
7. I feel the house chores are more equally divided among household members.	901	417 (46.3)	158 (17.5)	235 (26.1)	91 (10.1)
8. I've had time to enjoy personal interests or hobbies.	901	302 (33.5)	271 (30.1)	282 (31.3)	46 (5.1)
9. I've been having poor appetite.	903	643 (71.2)	123 (13.6)	129 (14.3)	8 (0.9)
10. I've been overeating.	902	422 (46.8)	115 (12.8)	289 (32.0)	76 (8.4)
11. I've been feeling tired or having little energy.	900	494 (54.9)	160 (17.8)	224 (24.9)	22 (2.4)
12. I've been feeling worried.	901	318 (35.3)	178 (19.8)	321 (35.6)	84 (9.3)
13. I've been feeling down.	901	410 (45.5)	189 (21.0)	260 (28.8)	42 (4.7)
14. I've had trouble falling or staying asleep.	899	478 (53.2)	172 (19.1)	209 (23.3)	40 (4.4)
15. I've been feeling lonely	900	562 (62.4)	134 (14.9)	184 (20.4)	20 (2.2)
16. I've had trouble relaxing.	895	561 (62.7)	153 (17.1)	162 (18.1)	19 (2.1)
17. I feel the house chores are less equally divided among household members.	897	548 (61.1)	141 (15.7)	150 (16.7)	58 (6.5)
18. I've become easily annoyed or irritable.	898	371 (41.3)	176 (19.6)	288 (32.1)	63 (7.0)

Valid (*n*) = Number of responses in each question.
